# Aging-Dependent Repair Performance and Interfacial Durability of New–Aged Waterproof Membrane Systems

**DOI:** 10.3390/polym18020163

**Published:** 2026-01-07

**Authors:** Chao Zhang, Xian Li, Xiaopeng Li, Longjiang Yang, Guojun Sun, Xingpeng Ma

**Affiliations:** 1 China Electronics Engineering Design Institute Co., Ltd., Beijing 100142, China; zhangchaotim@163.com; 2 China Electricity Council, Beijing 100761, China; 3 China Construction Sixth Engineering Bureau Co., Ltd., Tianjin 300012, China; lxp1150@163.com; 4 Faculty of Architecture, Civil and Transportation Engineering, Beijing University of Technology, Beijing 100124, China; maxingpeng@emails.bjut.edu.cn

**Keywords:** durability, heterogeneous combination, interface performance, peel strength, waterproof membrane

## Abstract

Waterproofing systems frequently experience performance degradation during long-term service due to material aging and structural deformation, thereby necessitating localized repair interventions. The bonding interface between newly applied and existing membrane materials is a critical determinant of repair effectiveness. In this study, the aging-dependent repair performance of three representative waterproof membrane systems was systematically investigated using peel strength testing, low-temperature flexibility assessment, and interfacial morphology analysis under thermal–oxidative aging for 2, 5, 14, and 28 days. The results demonstrate that the homogeneous repair system based on ultra-thin reinforced self-adhesive polymer-modified bituminous membranes exhibits superior overall performance, maintaining the highest peel strength with only minor degradation even after 28 days of accelerated aging. In contrast, the polymeric butyl self-adhesive membrane subjected to homogeneous repair exhibited rapid adhesion degradation after 14 days, whereas the heterogeneous repair system showed improved stability during intermediate aging stages. Low-temperature flexibility testing further revealed that root-resistant bituminous membranes exhibited a slower aging rate, with a cracking temperature increase of 7 °C after 28 days, compared to a 10 °C increase observed for ultra-thin self-adhesive membranes. These quantitative findings provide clear guidance for the selection of appropriate repair membrane systems under varying aging conditions in waterproofing engineering, particularly for maintenance and rehabilitation applications.

## 1. Introduction

**Figure 1:**
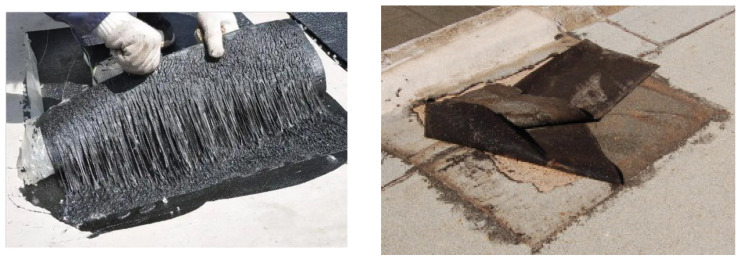
Case of Waterproof Membrane Repair Failure.

Building waterproofing is a critical factor in ensuring the durability and structural safety of buildings, with waterproof membranes serving as essential materials widely applied in roofs, basements, and foundations. Their primary function is to protect buildings from environmental factors such as rainfall, solar radiation, and wind erosion, thereby mitigating concrete cracking and reinforcing steel corrosion and ensuring structural integrity [1]. However, during prolonged service, waterproof membranes inevitably undergo degradation phenomena such as cracking and mechanical damage, which may ultimately result in leakage. Consequently, timely repair or replacement becomes essential to maintain waterproofing performance [2]. Figure 1 presents a representative example of waterproof membrane repair failure. In recent years, composite waterproofing systems have progressively replaced traditional single-layer membranes, significantly enhancing overall waterproofing performance while providing new technical solutions for repairing aged membranes. In this context, the overlap configuration and interfacial bonding between new and existing membranes become the key factors governing repair success.

The long-term stability of waterproofing systems is largely governed by the compatibility between different constituent materials. Compatibility refers to the ability of different waterproof materials to maintain stable interfacial bonding and achieve synergistic waterproofing performance under combined service conditions [3]. In general, greater similarity in chemical composition and molecular structure between materials results in improved compatibility. Conversely, pronounced disparities in these properties may induce adhesion failure, delamination, interfacial debonding, blistering, and edge lifting, or even trigger adverse physicochemical reactions, ultimately leading to the failure of the waterproofing layer. Therefore, systematic investigation of the compatibility and interfacial bonding performance between different waterproof materials is essential for improving the durability and reliability of building waterproofing systems.

In recent years, extensive studies have been conducted worldwide on the composite application and compatibility of waterproof materials. Chen et al. [4] investigated the improper combination of cement-based crystalline waterproof materials (CCCW) and SBS-modified bituminous membranes in basement engineering, highlighting the risks arising from insufficient material compatibility. Garrido et al. [5] reported that peel failure during lap performance testing between new and aged SBS membranes predominantly occurred on the aged membrane side, indicating that aging significantly degrades interfacial performance. Polyurethane–bitumen fusion materials exhibit advantages in accommodating structural deformation and enhancing tensile and tear resistance owing to their high elongation and excellent interfacial adhesion [6]. Furthermore, Lopes et al. [7] demonstrated that, in PVC membrane repair, hot-air welding may induce material damage and reduce deformation capacity, whereas solvent bonding results in superior overall mechanical performance.

The performance of the adhesive layer in waterproof membranes plays a decisive role in determining the overall waterproofing performance. With regard to adhesive layer performance, previous studies have primarily focused on the effects of construction-related parameters—such as bonding temperature, adhesive layer thickness, and adhesive type—on bonding effectiveness. On this basis, corresponding construction strategies have been proposed to ensure reliable adhesion under various environmental conditions [8,9]. Li [3] systematically analyzed the compatibility of composite waterproof materials in different application scenarios, emphasizing the importance of considering both physical and chemical interactions. Tan and Zhou et al. [10,11] reported a pronounced interfacial effect between polyurethane coatings and bituminous materials, leading to a reduction in peel strength. Wang et al. [12] elucidated the mechanism of mechanical property degradation in SBS membranes after seasonal aging from a microstructural perspective, while Han et al. [13] further demonstrated that composite self-adhesive bituminous membranes exhibit excellent mechanical properties and impermeability.

Meanwhile, in practical engineering applications, the combined use of different waterproof materials is widespread. For instance, rubber-, bitumen-, and PVC-based membranes are commonly applied in roofs, basements, and foundations under damp environmental conditions. Re-coating existing membranes with additional waterproof layers can significantly enhance overall protective performance [14,15]. In localized areas such as wall corners and pipe penetrations, the combined use of polymer membranes and waterproof coatings can effectively enhance waterproofing performance under complex conditions [16]. Moreover, Xiao et al. [17] proposed a novel acrylic waterproof coating that exhibits good compatibility and aging resistance when combined with bituminous membranes, thereby effectively extending the service life of the waterproofing system. A large-scale survey by Beer et al. [18] showed that long-term service of PVC roofing membranes results in a significant decrease in low-temperature flexibility and elongation at break. Aging is primarily induced by environmental factors such as oxygen, heat, and ultraviolet radiation, which severely compromise material durability [19].

In summary, existing studies have made substantial progress in the composite application of waterproof membranes and coatings, the factors influencing interfacial bonding performance, and the aging mechanisms of waterproofing materials. However, current studies lack a systematic investigation into the compatibility of different types of waterproof membranes during aging, particularly the degradation mechanisms of bonding performance at lap joints formed between aged and new membranes. In contrast to previous studies that primarily focused on material compatibility under unaged conditions or on the aging behavior of single waterproofing materials [4,5,6,7,10,11,12,13], the present study systematically investigates the aging-dependent repair performance of interfaces formed between new and aged waterproof membranes. By combining controlled thermal–oxidative aging with repair-oriented peel strength testing, low-temperature flexibility evaluation, and interfacial morphology analysis, this work captures the evolution of interfacial bonding behavior throughout the repair lifecycle. The results provide both mechanistic insight into interfacial degradation during aging and practical guidance for the selection of appropriate repair membrane systems under different aging conditions, thereby contributing to the improvement of long-term durability and service life of waterproofing systems.

## 2. Experimental

### 2.1. Materials

Waterproof membranes manufactured by Dongfang Yuhong (Beijing, China) were selected as the experimental materials. The selected membrane types included ARC polymer-modified bituminous chemical root-penetration-resistant waterproof membrane (RRBM), FXZ-150 polymeric butyl self-adhesive waterproof membrane (PBM), and ShuiLidun ultra-thin reinforced self-adhesive polymer-modified bituminous waterproof membrane (URBM). The detailed specifications of these membranes are summarized in Table 1.

**Figure 2:**
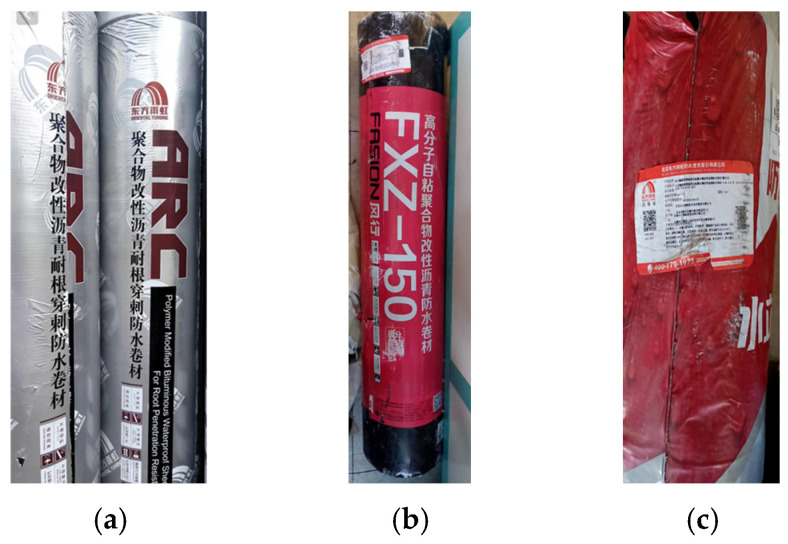
Waterproofing Membranes of Different Materials: (**a**) RRBM; (**b**) PBM; (**c**) URBM.

The three types of membranes are shown in Figure 2.

### 2.2. Experimental Program

(1) Specimen Preparation

**Figure 3:**
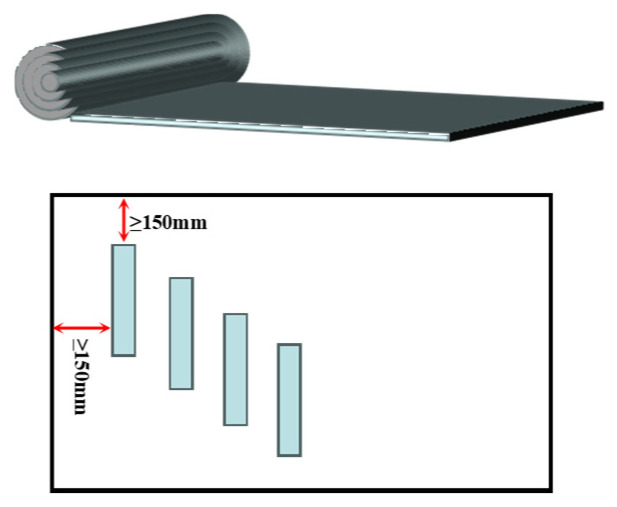
Specimen Cutting Diagram.

Specimen preparation was conducted in strict accordance with the Test Methods for Waterproof Membranes in Buildings (GB/T 328.1-2007) [20]. The detailed preparation procedure was as follows:

(1) Membranes subjected to different aging durations were uniformly cut along the width direction to ensure sampling consistency.

(2) To avoid the influence of uneven edge aging or damage, all specimens were cut at least 150 mm away from the membrane edge.

(3) During cutting, the long side of each specimen was aligned parallel to the longitudinal direction of the membrane to ensure that the stress orientation during testing corresponded to actual service conditions.

(4) For compatibility evaluation, all specimens were prepared with uniform dimensions of 50 mm × 200 mm, which are suitable for peel strength testing, as illustrated in Figure 3.

All specimens were prepared under identical environmental and operational conditions to ensure experimental reproducibility.

(2) Repair Treatment

To investigate the repair performance of different waterproof membranes, compatibility tests were systematically conducted under various aging durations. Here, “compatibility” specifically refers to the ability of new and aged waterproof membranes to form a stable and durable bonded interface during repair, as assessed by peel resistance, failure mode, and interfacial integrity under aging conditions. Membrane specimens subjected to thermal-oxidative aging for 2, 5, 14, and 28 days were subsequently repaired using different types of new waterproof membranes. These aging durations were selected to simulate different stages of material degradation under accelerated laboratory conditions. Short-term aging represents the initial degradation stage following repair, whereas longer aging durations correspond to progressively advanced deterioration processes. Previous studies comparing laboratory-accelerated aging with outdoor environmental exposure have demonstrated that extended laboratory aging can effectively reproduce the physicochemical and mechanical degradation characteristics observed under long-term field aging conditions [21].

**Figure 4:**
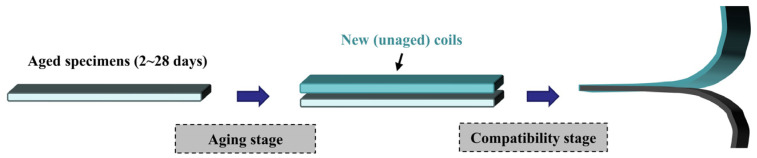
Repair Treatment Flowchart.

Prior to repair, the surfaces of the aged specimens were thoroughly cleaned, after which new membrane strips (50 mm × 200 mm) were bonded onto the aged substrates. Bonding pressure, bonding width, and lap length were strictly controlled to ensure consistent repair conditions. The repaired specimens were subsequently conditioned in a standard testing environment (23 ± 2 °C, 50 ± 10% relative humidity) for 24 h to allow complete curing of the bonding interface, after which peel tests were performed to ensure the reliability of the peel strength data. The experimental procedure for the repair treatment is schematically illustrated in Figure 4. In addition, low-temperature flexibility tests were conducted on aged specimens to quantify performance degradation induced by aging.

(3) Testing Methods

**Figure 5:**
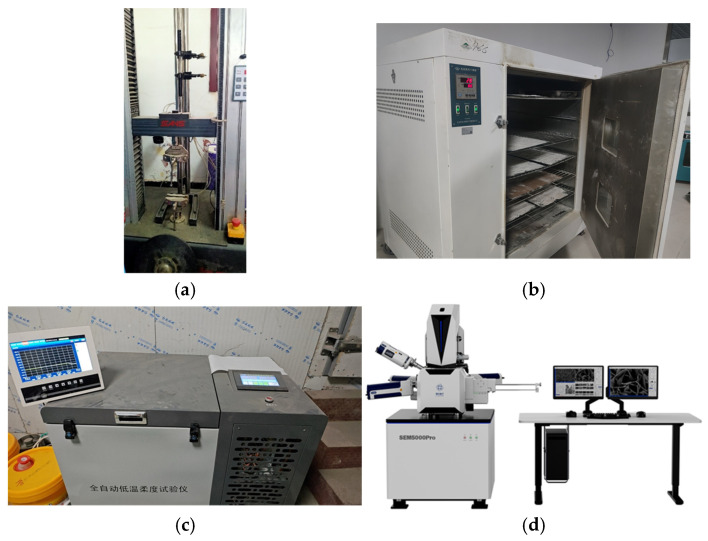
Test Equipment: (**a**) Universal Testing Machine, (**b**) Film Oven, (**c**) Low-Temperature Flexibility Testing Instrument, (**d**) SEM5000Pro Field Emission Scanning Electron Microscope.

The seam peel performance test was conducted in accordance with Test Methods for Waterproof Membranes in Buildings (GB/T 328.20-2007) [20]. The test was performed using a universal testing machine, with peel specimens measuring 200 mm × 50 mm and a seam width of 100 mm. The fixture spacing was set to 100 mm, and the crosshead displacement rate during testing was maintained at 100 mm/min. For the low-temperature flexibility test, an automatic low-temperature flexibility testing apparatus was employed. The testing apparatus is shown in Figure 5.

## 3. Results and Discussion

### 3.1. Appearance and SEM Analysis

**Figure 6:**
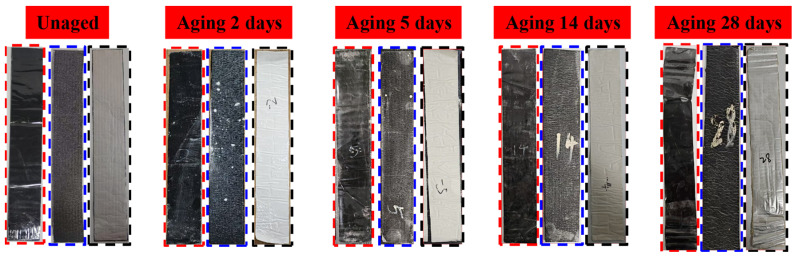
Comparison of Appearance Between Unaged and Aged Specimens.

A comparison between unaged specimens and those subjected to coupled aging is presented in Figure 6. In the figure, red dashed lines represent RRBM, blue dashed lines represent PBM, and black dashed lines represent URBM. Pronounced differences in appearance were observed between the unaged specimens and those subjected to coupled aging. The surfaces of the unaged specimens were smooth, exhibiting high reflectivity and uniform coloration. With increasing aging duration, the specimens gradually developed surface wrinkling, accompanied by increased surface roughness, noticeable gloss fading, and localized yellowing. Dispersed spots attributable to bitumen oxidation were also observed [22]. In addition, thermal expansion caused the edges of the aged specimens to become more rounded, resulting in an overall change in specimen geometry.

**Figure 7:**
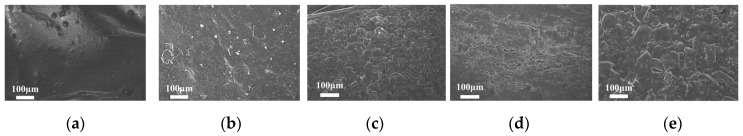
Scanning Electron Microscope (SEM) Images Before and After Aging: (**a**) Unaged, (**b**) Aged 2 Days, (**c**) Aged 5 Days, (**d**) Aged 14 Days, (**e**) Aged 28 Days.

Figure 7 presents SEM images of URBM specimens in the unaged condition and after 2, 5, 14, and 28 days of aging. For SEM observation, membrane specimens were sectioned into small pieces from the bonded interfacial region after peel testing. The samples were dried at room temperature to remove surface moisture and subsequently sputter-coated with a thin gold layer to ensure electrical conductivity. SEM observations were conducted using a scanning electron microscope under high-vacuum conditions. SEM images were acquired at low magnification to capture the overall interfacial morphology, with the corresponding scale bar indicated in each image. These images reveal a continuous evolution of surface morphology with increasing aging duration. In the unaged state, the membrane surface appears relatively smooth, with scattered intrinsic pores. As aging progresses, the surface becomes increasingly rough, accompanied by granular precipitates and irregular convex–concave microstructures. These morphological changes are primarily attributed to the volatilization of light components and polymerization–oxidation reactions within the bituminous matrix [23]. Although the formation of a dense granular surface layer may slightly retard further aging, it generally indicates increased brittleness and reduced flexibility, which macroscopically manifests as a gradual deterioration in waterproofing performance and durability.

### 3.2. Low-Temperature Flexibility Analysis

Low-temperature flexibility tests of the self-adhesive membranes were conducted in accordance with the requirements of Self-adhesive Polymer-modified Bitumen Waterproof Membranes (GB 23441-2009) [24]. The crack temperatures obtained from low-temperature flexibility testing for URBM and RRBM specimens, in both the unaged state and after different aging durations, are summarized in Table 2.

As shown in Table 2, the low-temperature flexibility of polymer-modified bituminous waterproof membranes progressively deteriorated with increasing aging duration. The RRBM exhibited superior low-temperature flexibility and aging resistance compared with the URBM. After 28 days of aging, the cracking temperature of the RRBM specimen increased moderately from −35 °C to −28 °C (a 7 °C change), whereas that of the URBM specimen increased markedly from −28 °C to −18 °C (a 10 °C change). The improved aging resistance of the RRBM is mainly attributed to the presence of chemical root-resistant additives, which inhibit oxidative degradation and delay flexibility loss. Accordingly, the RRBM exhibited a slower aging rate in low-temperature flexibility than the URBM. This behavior is primarily attributed to the incorporation of chemical root-resistant additives and stabilizing agents within the modified bituminous matrix, which effectively inhibit oxidative reactions and retard the volatilization of light components during thermal-oxidative aging, thereby delaying flexibility degradation. In addition to chemical effects, structural characteristics may also contribute to the enhanced aging resistance of the root-resistant membrane. Its relatively greater thickness and more stable modified bitumen system can reduce oxygen diffusion and thermal penetration, resulting in lower aging sensitivity [25,26]. Nevertheless, chemical stabilization is considered the dominant factor, whereas structural differences play a secondary role.

### 3.3. Compatibility Analysis

**Figure 8:**
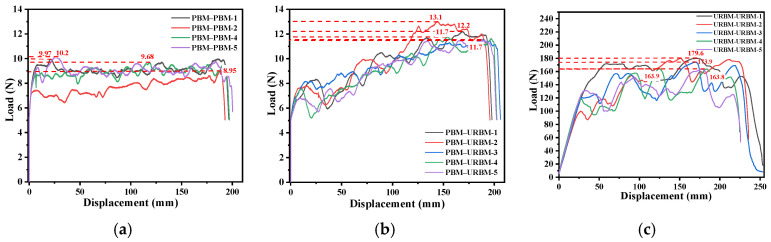
Peel Load–Displacement Curves of Repaired Unaged Waterproof Membranes. (**a**) PBM-PBM; (**b**) PBM-URBM; (**c**) URBM-URBM.

Unaged waterproof membrane specimens were repaired by bonding, and peel tests were conducted to evaluate their peel behavior and interfacial compatibility. Figure 8 presents the peel load–displacement curves of the repaired unaged specimens. For the PBM bonded to itself (Figure 8a), the peel load ranged from approximately 9 to 10 N. Although relatively high peel loads were observed at the initial stage, a rapid decrease in load occurred with increasing displacement, indicating limited resistance to progressive peeling. This behavior suggests that failure was dominated by interfacial adhesive failure, reflecting insufficient interfacial toughness.

In contrast, the PBM–URBM combination (Figure 8b) exhibited a more gradual increase in load, particularly at larger displacement levels. This response indicates enhanced interfacial deformation capacity and a transition toward mixed adhesive–cohesive failure. Moreover, the homogeneous URBM–URBM combination (Figure 8c) demonstrated the highest peel performance, with the load increasing steadily and remaining at a high level even at large displacements. This behavior is characteristic of cohesive or mixed failure within the adhesive or bituminous layer, indicating superior interfacial compatibility and bonding strength. Overall, among the unaged specimens, the URBM exhibited superior absolute peel strength compared with the PBM.

**Figure 9:**
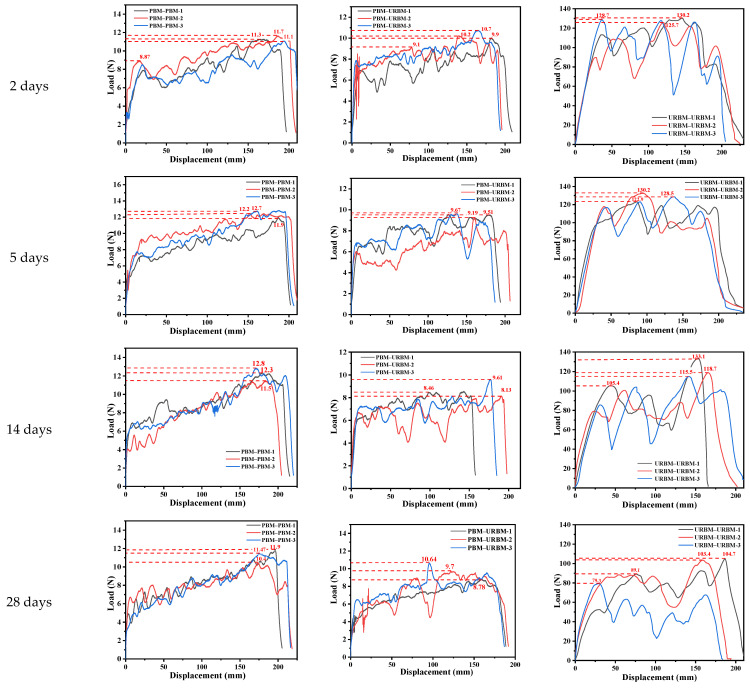
Peel Load–Displacement Curves of Repaired Waterproof Membranes After 2–28 Days of Aging.

Specimens subjected to different aging durations (2, 5, 14, and 28 days) were subsequently repaired and tested to evaluate the evolution of peel behavior under aging conditions. Figure 9 presents the peel load–displacement curves of the repaired specimens after aging. For the PBM–PBM repair system (Figure 9), relatively high peel loads were observed after short-term aging (2 days); however, the peel load decreased markedly with increasing aging duration, particularly after 14 and 28 days [26]. This rapid decline indicates poor aging resistance and a progressive shift toward interfacial adhesive failure as the dominant failure mode.

For the PBM–URBM repair system (Figure 9), moderate peel loads were observed at the early aging stage, with only minor variations between 2 and 5 days. After 14 days of aging, a gradual reduction in peel load was observed; however, the degradation rate was slower than that of the PBM–PBM system. This behavior indicates improved relative peel performance, reflected by enhanced retention of interfacial bonding with increasing aging duration. In contrast, the homogeneous URBM–URBM repair system (Figure 9) exhibited stable peel behavior throughout all aging stages. Even after 14 and 28 days of aging, the peel load remained high with only minor reductions, indicating excellent aging resistance and stable cohesive or mixed failure modes.

**Figure 10:**
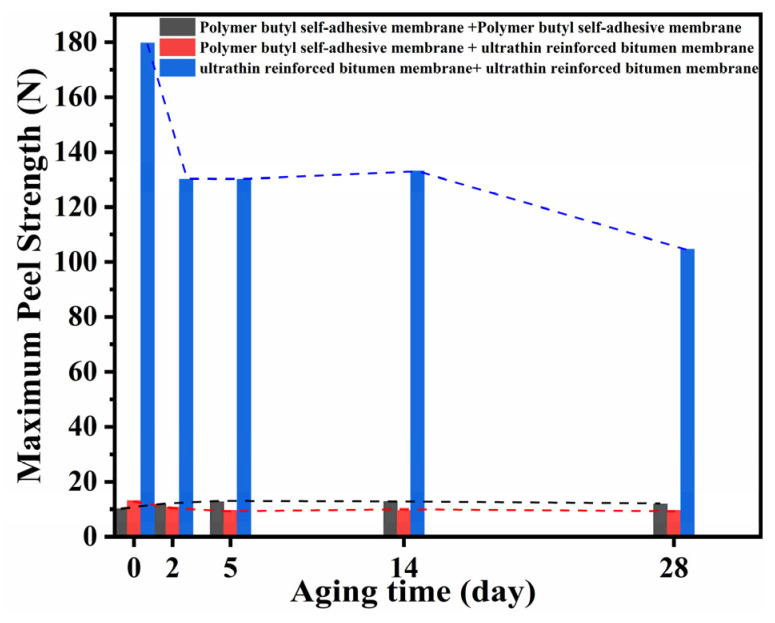
Variation in Peel Strength of Specimens Under Different Aging Durations.

Figure 10 summarizes the evolution of peel strength under different aging conditions. Although the peel strength of all membrane combinations decreased with aging, clear distinctions were observed between absolute peel strength and relative performance retention. The homogeneous URBM–URBM system maintained the highest absolute peel strength throughout the aging process, demonstrating superior long-term durability. Meanwhile, the heterogeneous repair system exhibited comparatively better relative performance in terms of peel strength retention during intermediate aging stages. From an engineering repair perspective, the PBM–URBM combination provides a balanced solution by offering acceptable absolute peel strength together with improved aging stability and repair adaptability.

### 3.4. Peel Interface

**Figure 11:**
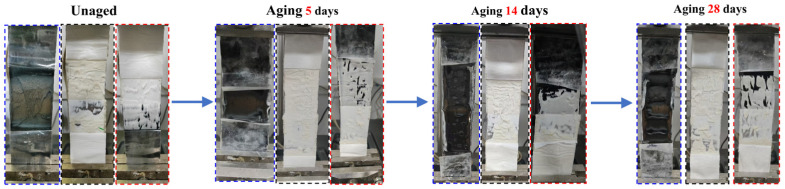
Interfacial Peel Morphologies at Different Aging Durations.

Figure 11 illustrates the interfacial morphology evolution of three membrane combinations (indicated by blue, black, and red boxes, respectively) at four stages: unaged, and after 5, 14, and 28 days of aging. The blue box corresponds to the URBM–URBM combination, the black box corresponds to the URBM–PBM combination, and the red box corresponds to the PBM–PBM combination.

In the unaged state, the interfaces of all three membrane combinations appeared clear and intact, with smooth surfaces and no apparent defects, indicating favorable initial bonding conditions. After 5 days of aging, discernible changes began to emerge at the interfaces. For some PBM–PBM specimens, as well as PBM–URBM specimens, fine surface cracks initially appeared, accompanied by localized debonding, suggesting a decline in interfacial integrity.

After 14 days of aging, degradation intensified markedly, with all three membrane interfaces exhibiting pronounced deterioration. Surface cracks expanded and deepened, while peeling and wrinkling became increasingly prevalent. The originally well-defined interfacial structures became increasingly indistinct. In particular, in URBM–URBM and PBM–PBM repair systems, large-scale coating detachment occurred in localized regions, indicating a substantial loss of bonding performance.

After 28 days of aging, interfacial degradation became increasingly severe. Most specimen surfaces exhibited extensive rupture, peeling, and curling, with interfacial layers becoming nearly indistinguishable, reflecting severe loss of material integrity. Among the three systems, the regions marked with red frames also exhibited pronounced degradation but retained relatively greater surface continuity; by contrast, specimens marked with blue and black frames displayed severely fractured interfacial structures, indicating comparatively weaker repair performance.

## 4. Conclusions

Based on peel testing, low-temperature flexibility evaluation, and interfacial morphology analysis, this study elucidates the aging-dependent repair behavior of different waterproof membrane combinations under thermal-oxidative aging. The main conclusions are as follows:

(1) Aging significantly governs the durability of repaired membrane interfaces. Although all membrane combinations exhibited a decline in peel resistance with increasing aging duration, homogeneous repair systems based on ultra-thin reinforced self-adhesive bituminous membranes showed superior resistance to aging-induced degradation, maintaining stable interfacial bonding even under advanced aging conditions.
(2) Low-temperature flexibility serves as an effective indicator of material aging resistance. Root-resistant membranes exhibited slower aging rates than ultra-thin self-adhesive membranes, highlighting the beneficial role of chemical stabilizing additives and a more robust material system in delaying thermal-oxidative degradation.
(3) Interfacial degradation during aging is governed by microstructural evolution. The transition from smooth surfaces to granular and reticular morphologies under prolonged aging leads to increased brittleness and reduced interfacial integrity, which underpins the observed macroscopic deterioration in bonding performance.
(4) From an engineering repair perspective, material selection should be tailored to the aging condition of existing membranes. For lightly aged substrates, homogeneous membrane repair provides optimal bonding strength and durability. For moderately to severely aged membranes, heterogeneous repair using polymer butyl self-adhesive membranes combined with bituminous membranes offers improved adaptability and interfacial stability, making it a practical solution for long-term waterproofing repair applications.

## Figures and Tables

**Table 1:** Characteristics of SBS-modified asphalt.

Membrane Type	Thickness (mm)	Low-Temperature Flexibility (°C)	Heat Resistance	Initial Elongation	Structural Feature	Polymer Matrix	Reinforcement
Root-resistant bituminous membrane (RRBM)	4.0	-No cracking at −25 °C	No flowing or dripping at 105 °C for 2 h	Low	Thick modified bitumen layer	SBS-modified bitumen + Chemical Root Inhibitors	Pyramidal polyester felt
Polymeric butyl self-adhesive membrane (PBM)	1.5	-No cracking at −25 °C	No flowing or dripping at 80 °C for 2 h	High	Flexible polymer-based adhesive	Butyl Rubber (IIR) + Polyisobutylene	None
Ultra-thin reinforced bituminous membrane (URBM)	0.8	-No cracking at −25 °C	No flowing or dripping at 70 °C for 2 h	Moderate	Reinforced, thin adhesive layer	SBS-modified bitumen	Glass fiber

**Table 2:** Cracking temperature for low temperature flexibility test.

Ageing Time (Day)		0	2	5	14	28
Cracking temperature (°C)	RRBM	−35	−33	−32	−32	−28
	URBM	−28	−25	−24	−22	−18

## Data Availability

The original contributions presented in this study are included in the article. Further inquiries can be directed to the corresponding authors.
